# Single-cell lipidomic analysis of the epithelial-mesenchymal transition using mass spectrometry imaging

**DOI:** 10.1016/j.isci.2026.115847

**Published:** 2026-04-21

**Authors:** Ellen Marie Botne Quinsgaard, Marco Giampà, Veronica With Andreassen, Sebastian Krossa, Anna Nordborg, Jakub Idkowiak, Johannes V. Swinnen, Siver Andreas Moestue

**Affiliations:** 1 Department of Clinical and Molecular Medicine, Norwegian University of Science and Technology (NTNU), 7491 Trondheim, Norway; 2 KU Leuven, Department of Cellular and Molecular Medicine, Laboratory of Applied Mass Spectrometry (LAMaS), 3000 Leuven, Belgium; 3 VIB, VIB Technologies, Metabolomics Core Facility Leuven, 3000 Leuven, Belgium; 4 Department of Circulation and Medical Imaging, Norwegian University of Science and Technology (NTNU), 7491 Trondheim, Norway; 5 Central Staff, St.Olavs Hospital HF, 7006 Trondheim, Norway; 6 Department of Biotechnology and Nanomedicine, SINTEF Industry, 7034 Trondheim, Norway; 7 Laboratory of Lipid Metabolism and Cancer, Department of Oncology, Leuven Cancer Institute (LKI) and Leuven Institute for Single Cell Omics (LISCO), KU Leuven, 3000 Leuven, Belgium; 8 Department of Pharmacy, Nord University, 8049 Bodø, Norway

**Keywords:** Biological sciences, Cell biology, Lipidomics

## Abstract

The epithelial-mesenchymal transition (EMT) is a metastasis-promoting process whose heterogeneity has been extensively studied at a gene expression level. EMT involves reprogramming of lipid metabolism; however, there has been little focus lipid level heterogeneity. Here, we use mass spectrometry imaging (MSI) to measure glycerophospholipids at the single-cell level during EGF-induced EMT in MDA-MB-468 breast cancer cells. Cells undergoing EMT had reduced levels of PA, PS, PE, and PI-species and increased levels of PG-species and LPI (18:0). Multivariate analysis on the spatially resolved MSI-data revealed a heterogeneous metabolic response. Lipid levels were particularly affected by cell organization, as dispersed cells were more “EMT-like” than cohesive cells. The fraction of dispersed cells increased during EMT, indicating that pathways regulating adhesion and motility also regulate lipid metabolism. Gene expression analysis verified that EMT affected genes involved in glycerophospholipid biosynthesis. This work demonstrates heterogeneous regulation of glycerophospholipids in cancer cell populations undergoing EMT.

## Highlights

• The epithelial-mesenchymal transition (EMT) is a process that promotes metastasis
• EMT caused extensive regulation in phospholipid levels and biosynthetic genes
• Single-cell measurements revealed heterogeneous lipid level response
• Cell organization matters: dispersed cells had EMT-like lipid profiles

## Graphical abstract

**Figure:**
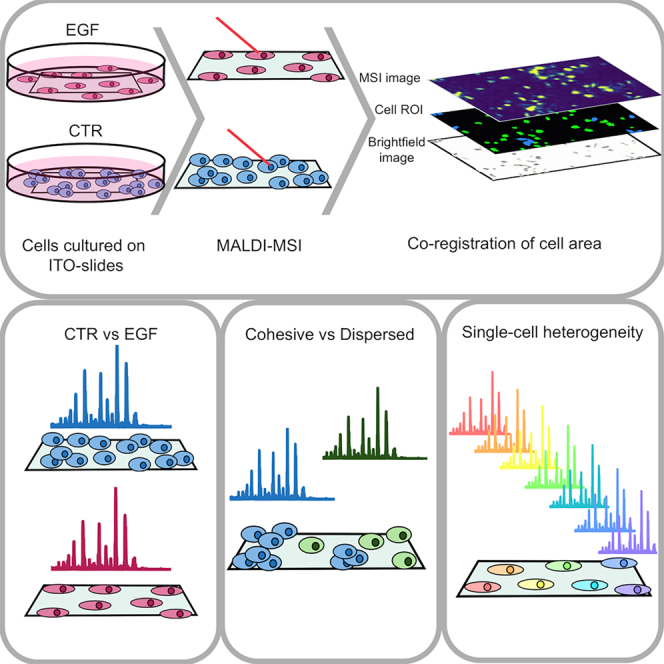

## Introduction

The epithelial-mesenchymal transition (EMT) is a process wherein epithelial cells take on mesenchymal features. The process has been found to occur during wound healing, fetus-development and in cancer where EMT has been found to promote metastasis.^1^ During EMT, metabolic rearrangements cause a shift in energy-usage away from cell proliferation and toward cell migration. This allows the cells to disseminate into foreign tissues, where they regain their proliferative abilities through the inverse process of mesenchymal-epithelial transition (MET). Besides the link to metastasis, EMT has also been found to promote therapy resistance.^1^^,^^2^ Hence, there is a need to understand the molecular events involved in EMT in order to counter its negative effects in cancer.

Among the many changes that occur during EMT are alterations in lipid metabolism. The transcription factors Twist1 and Snai1 are strong EMT-inducers that have been found to have additional roles as regulators of fatty acid oxidation and lipolysis, respectively.^3^^,^^4^^,^^5^ Glycerophospholipids are one of the main components of the cell membrane. They consist of two acyl chains bound to a head group via a glycerol backbone.^6^ Switching out the headgroups alters the properties of glycerophospholipids. Classes include phosphatidylcholine (PC) and phosphatidylethanolamine (PE), that are neutral, and phosphatidic acid (PA), phosphatidylglycerol (PG), phosphatidylinositol (PI), and phosphatidylserine (PS) that are negative.^7^

**Figure 1:**
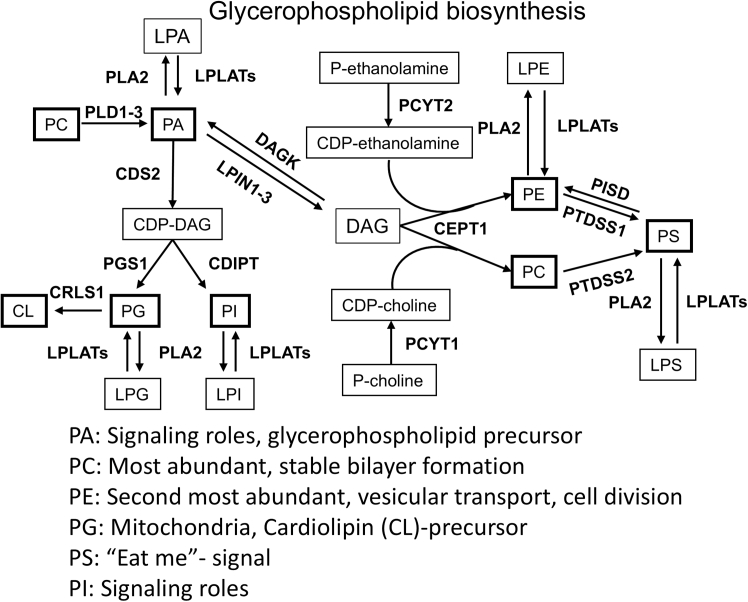
Overview glycerophospholipid biosynthesis^10^^,^^12^

PC and PE are the two most abundant glycerophospholipids, making up 41–57 and 17–38 mol %, respectively, of total glycerophospholipids.^8^ PC has a cylindrical shape that mediates its organization into stable bilayer formation, while PE has a smaller head group that gives it a conical shape.^8^ The conical shape of PE promotes membrane curvature, which is necessary for cell division and vesicular transport.^7^^,^^8^^,^^9^ PE and PC are both synthesized by the addition of their respective headgroups to diacylglycerol (DAG), and both can have their headgroups exchanged to produce PS (Figure 1).^10^ PS is primarily localized in the ER or in the inner leaflet of the plasma membrane. During apoptosis PS relocalizes to the outer leaflet, causing the apoptotic cell to be identified and removed by phagocytes.^11^ Intracellularly, PS binds and recruits proteins. Some proteins bind preferentially to PS, while others bind non-specifically to the headgroups of charged lipids including PS.^11^

Like PS, PG can also act as a protein binding site and has been shown to participate in protein kinase C (PKC)-activation. PG is primarily localized in the mitochondria, where it acts as a precursor to cardiolipin (CL).^13^^,^^14^ The PI head group is an inositol ring which can be phosphorylated on multiple sites, and phosphorylated PI has multiple, extensively reviewed signaling roles.^15^^,^^16^^,^^17^ Of particular note is its role in PI3K-signaling.^18^ PG and PI biosynthesis are both downstream of CDP-DAG (Figure 1).^10^ Finally, PA is the glycerophospholipid with the smallest head group and acts as precursor to the others (Figure 1).^10^ PA acts as a signaling molecule, activating kinases including 3-phosphoinositide-dependent protein kinase-1 (PDK1), mitogen-activated protein kinase (MAPK), and the mammalian target of rapamycin (mTOR).^13^^,^^19^

Levels of glycerophospholipids have been found to differ in breast cancer cell lines with different metastatic potential.^20^^,^^21^ For instance, PA-levels are higher in metastatic MDA-MB-231 than non-metastatic T-47D.^20^ Saturation of acyl chains is also of interest in EMT as cells with high motility tend to have lower saturation of membrane phospholipids and higher membrane fluidity.^20^^,^^21^ Reduced membrane fluidity has in turn been found to inhibit EMT.^22^^,^^23^ Saturation of glycerophospholipids are adjusted by the exchange of acyl chains. Members of the phospholipase A2 (PLA2) family remove acyl chains before a lysophospholipid acyltransferase (LPLAT) adds a chain.^24^

Studying the molecular alterations associated with EMT is complicated by the heterogeneity of cell populations. Most cells in a population are in partial/transitioning states, positioned somewhere in the continuous spectrum from fully epithelial to fully mesenchymal.^25^^,^^26^^,^^27^^,^^28^ Disregarding the heterogeneity of EMT may obscure associations between phenotype and metabolic activity. As we unravel the importance of changing lipid composition in EMT, as well as the emerging clinical relevance of heterogeneous partial EMTs it seems prudent to investigate heterogeneity in EMT lipidomics.

Matrix-assisted laser desorption ionization mass spectrometry imaging (MALDI-MSI) is a method that can be used to measure lipids and other analytes in biological materials. It allows spatially resolved metabolic analyses, and the technology has the potential to open up new avenues within single-cell spatial omics. MALDI-MSI has mostly been used to study tissue samples, but may also be used to determine lipids in cells cultured *in vitro.*^29^ Furthermore, instruments with higher spatial resolution have made it possible to use the method to study single cells.^30^ Indeed new MALDI-MSI-based technologies are able to image lipids at the subcellular level.^31^

Here, we use MALDI-MSI to explore the metabolic changes that occur during EMT of the breast cancer cell line MDA-MB-468. We explore the heterogeneity of these changes at a single-cell level and identify how lipid-levels change when cells spread out by comparing “lonely” dispersed cells with cohesive groups of cells.

## Results

### Inducing EMT with EGF

**Figure 2:**
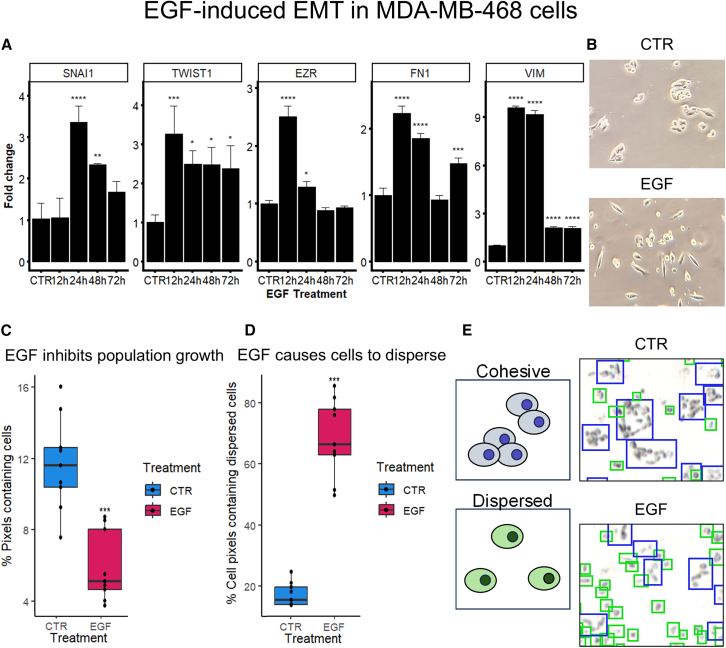
EGF induces EMT in MDA-MB-468 cells Comparison of untreated CTR samples vs. samples treated with 25 ng/mL EGF. (A) Gene expression changes measured by qPCR after 0, 12, 24, 48, and 72 h of EGF-treatment. Shows fold changes of the mesenchymal markers *EZR*, *FN1*, and *VIM*, as well as the EMT-transcription factors *SNAI1* and *TWIST1*. Error bars represent standard deviations. *N* = 3. *p* values calculated using Bonferroni-corrected, pairwise two-sample *t* tests vs. CTR. (B) Morphology of CTR cells and cells treated with EGF for 72 h. (C) Boxplot displaying percentage of pixels containing cells in MALDI-MSI image. (D) Boxplot displaying what percentage of cell-containing pixels contained dispersed single cells, rather than cohesive groups of cells. *N* = 9. The boxes represent the interquartile range (IQR) while the error bars represent Q1 –1.5∗IQR and Q3 +1.5∗IQR respectively. *p* values calculated using the two-sample *t* test. (E) Illustration and example of cohesive and dispersed cells. Significance levels: *p* < 0.05: “∗,” *p* < 0.01: “∗∗,” *p* < 0.001: “∗∗∗.”

EMT was induced by culturing MDA-MB-468 breast cancer cells with 25 ng/mL epidermal growth factor (EGF).^32^ EMT-induction was assessed by measuring the EMT-transcription factors *TWIST1* and *SNAI1*, as well as the mesenchymal biomarkers Vimentin (*VIM*) and Fibronectin1 (*FN1)* and the EMT-promoting Ezrin (*EZR*).^33^^,^^34^^,^^35^^,^^36^^,^^37^ Increased gene expression was observed in all these biomarkers by qPCR, indicating successful EMT-induction (Figure 2A). Transcriptional bursts were observed for all markers at 12 h or 24 h.^38^ In all replicates, we observed EGF-induced morphological changes. EGF-treated samples contained a mixture of spindle-like and round cells. This phenotype remained stable at 72 h, consistent with previous observations (Figure 2B).^32^^,^^39^

It was decided to measure lipid levels 72 h after EMT induction to capture the lipidomic effect on this stable, post-mitotic phenotype. For the MALDI-MSI-experiments cells were cultured on ITO-slides with or without EGF for 72 h. EGF has been found to inhibit proliferation and promote apoptosis in MDA-MB-468 cells.^32^^,^^40^^,^^41^ After performing MALDI-MSI, the pixels containing cells were registered. Control (CTR) samples had on average 11.7% cell-containing pixels, while an average of 5.9% of pixels contained cells in the EGF-samples. (Figure 2C) This is consistent with reduced proliferation and survival in EGF-stimulated cells. One property of EMT is that cells become less adherent and spread out as dispersed single-cells.^2^ In order to quantify changes in cell organization we measured the number of pixels containing cohesive groups or dispersed cells. MSI-pixels were categorized as either cohesive or dispersed by analyzing the corresponding brightfield image. In the EGF-treated cells, on average 68% of the cell-containing area was assigned to dispersed cells, whereas only 17% of control cells were classified as dispersed (Figures 2D and 2E).

### EMT induces changes in glycerophospholipid-levels

**Figure 3:**
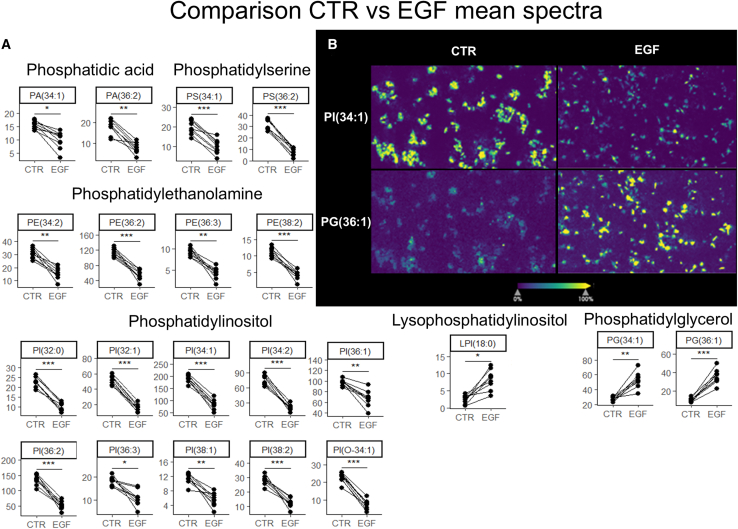
Comparison of lipid levels in CTR vs. EGF-treated samples (A) Paired dot plots comparing lipid levels in CTR vs. EGF-treated samples. Measured by MALDI-MSI. One mean spectrum extracted from each sample. Datapoints paired according to the round of matrix-application shared by samples of each treatment group. *p* values calculated using paired *t* tests and adjusted using the Bonferroni-approach. Significance levels: *p* < 0.05: “∗,” *p* < 0.01: “∗∗,” *p* < 0.001: “∗∗∗.” (B) MSI-map example of lipids PI(34:1) and PG(36:1) in one CTR sample and one EGF sample.

To determine the effects of EGF-induced EMT on lipid levels, MDA-MB-468 cells were measured by MALDI-MS. Cells were cultured on ITO-slides and treated with either EGF-containing (25 ng/mL) or EGF-free CTR media for 72 h. CTR and EGF-slides were paired up during shared rounds of matrix-application before MALDI-MSI. MSI-datasets were TIC-normalized, and peak picking was performed before cell-specific pixels were identified by spatial *k*-means clustering. One mean spectrum was extracted from each slide based on all pixels that contained cells. Peaks with minimum five times higher signal in cell spectra than cell-free spectra were defined as cell specific. Lipid identities were determined based on accurate mass. Lipid identities were determined for 29 out of 46 peaks of interest (Table S2). The treatment groups were compared using a paired *t* test, and the levels of 21 lipids were found to be significantly different between treatment groups. EGF reduced the levels of two PA-species, two PS-species, four PE-species, and ten PI-species, and increased the levels of two PG-species and lysophosphatidylinositol (18:0) (LPI(18:0)) (Figure 3A). Visual inspection of lipid maps showed heterogeneous lipid levels within samples which were explored further (Figure 3B).

### There is an overlap between lipids that change during EMT and lipids that differentiate between cohesive vs. dispersed cells

**Figure 4:**
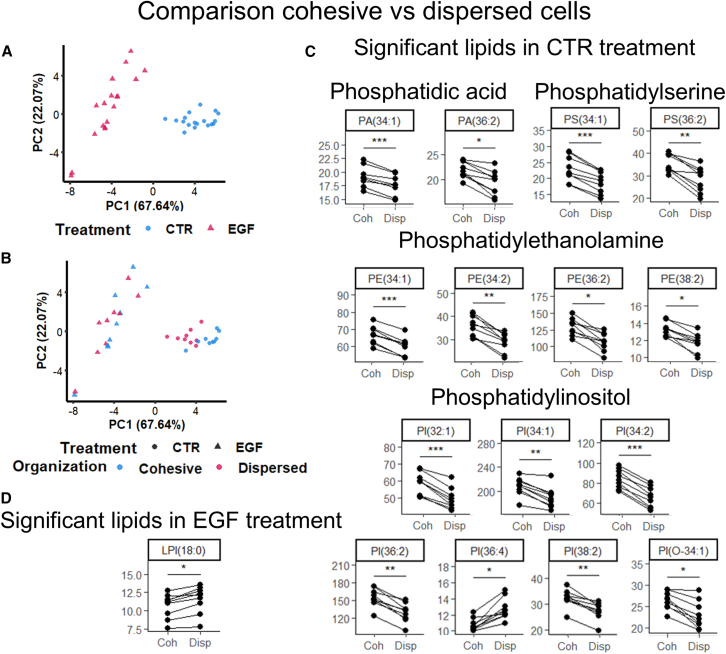
Comparison of lipid levels in cohesive vs. dispersed cells Two mean spectra were extracted from each sample: one from all pixels containing cohesive cells and one from all pixels containing dispersed cells. (A) PCA analysis colored according to treatment group. (B) PCA analysis colored according to organization of cells. (C) Paired dot plots displaying how lipid levels are affected by cell organization in CTR samples. (D) Paired dot plot displaying that LPI(18:0) is affected by cell organization in EGF samples. Datapoints paired according to which sample the mean spectra are extracted from. *p* values calculated using paired *t* tests and corrected using the Bonferroni approach. Statistical significance levels: *p* < 0.05: “∗,” *p* < 0.01: “∗∗,” *p* < 0.001: “∗∗∗.”

Since the cells were observed to spread out during EMT (Figure 2D), we were interested in determining whether dispersed single cells were more EMT-like than cohesive groups of cells. From each sample, we extracted two mean spectra: one based on all pixels containing cohesive cells and one based on all pixels containing dispersed cells. We included data from all samples in a PCA-analysis to evaluate the heterogeneity. This PCA-analysis showed clear separation of CTR and EGF samples on PC1 (Figure 4A). When colored according to organization, the PCA showed a separation of cohesive vs. dispersed cells within the CTR treatment. This separation was also in PC1, with dispersed cells closer to the EGF-cluster (Figure 4B). Spectra were paired according to which sample they were extracted from and compared using a paired *t* test. Within the CTR treatment, 14 lipids were found to be significantly different in cohesive vs. dispersed cells. Dispersed cells were found to have lower levels of two PA-species, two PS-species, three PE-species, and six PI-species, as well as higher levels of one PI-species (Figure 4C). In the EGF treatment, however, only LPI(18:0) was found differentiate cohesive and dispersed cells (Figure 4D).

The levels of PA(34:1), PA(36:2), PS(34:1), PS(36:2), PE(34:2), PE(36:2), PE(38:2), PI(32:1), PI(34:1), PI(34:2), PI(36:2), PI(38:2), and PI(O-34:1) were found to decrease in dispersed CTR cells, as well as to overall decrease in the EGF-treatment compared to the CTR. Conversely, LPI(18:0) increased both in dispersed EGF cells and in the EGF treatment compared to the CTR (Figures 4C and 3A). This indicates that the dispersed cells are strongly associated with the mesenchymal-like lipid profile.

The lipids PE(36:3), PI(32:0), PI(36:1), PI(36:3), and PI(38:1) decreased in the EGF treatment but not in dispersed CTR cells. PG(34:1) and PG(36:1) increased in the EGF-treatment but not in dispersed CTR cells. PI(36:4) increased in dispersed CTR cells but was unaffected by EGF-treatment (Figures 4C and 3A).

### Single-cell analysis reveals lipid-level heterogeneity of EMT response

**Figure 5:**
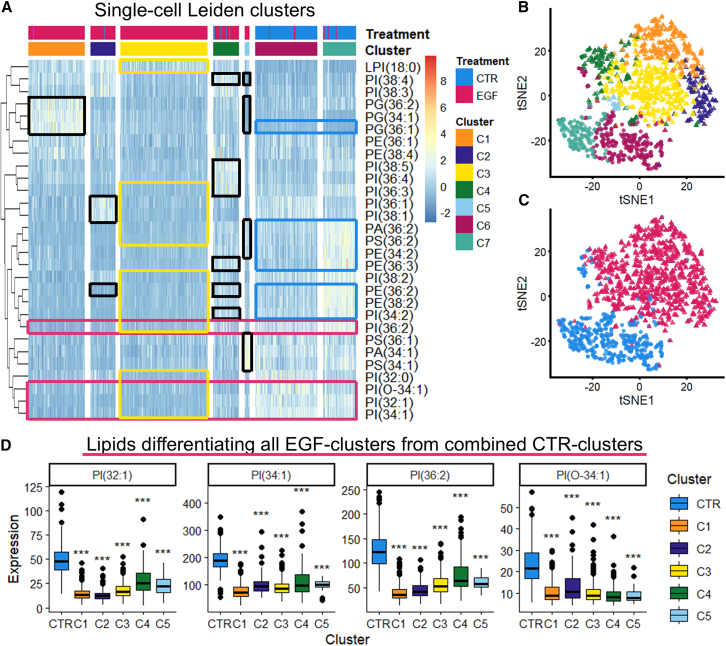
Single-cell clustering analysis performed on dispersed cells (A) Heatmap showing scaled lipid levels in clusters identified by Leiden clustering on a shared nearest neighbor graph. Black boxes: lipids differentiating individual EGF-majority clusters to remaining EGF-majority clusters. Blue boxes: lipids differentiating the two CTR-majority clusters C6 and C7. Magenta boxes: lipids differentiating each individual EGF-majority cluster from the CTR-majority clusters C6 and C7. All highlighted lipids fulfill the following: fold change > 1.5 or below 0.667, *p* value < 0.001, and non-overlapping IQR. (B) tSNE-embedding of single-cell lipid data with each datapoint colored according to cluster identity. (C) tSNE-embedding of single-cell lipid data colored according to treatment group. (D) Levels of differentiating lipids marked by magenta boxes in (A). The boxes represent the IQR while the error bars represent Q1 –1.5∗IQR and Q3 +1.5∗IQR respectively. *p* values calculated using two-sample *t* tests and corrected using the Bonferroni approach. Significance levels: *p* < 0.05: “∗,” *p* < 0.01: “∗∗,” *p* < 0.001: “∗∗∗.” See also Figures S1 and S2.

EMT is notorious for its heterogeneity, and various degrees and categories of EMT have been described at a gene expression level. We wanted to examine this heterogeneity at the phospholipid level. Dispersed single-cells were identified and cohesive cell groups were discarded to avoid effects from cell multiplets in the dataset. Spectra were extracted from each of the dispersed cells. Downstream analysis was performed on 446 (33.3%) CTR cells and 893 (66.7%) EGF cells. An initial PCA-analysis showed CTR- and EGF-stimulated cells as distinct but overlapping groups with separation along PC1 (Figure S1). Clustering was performed using the Leiden algorithm on a Shared Nearest Neighbor (SNN)-network. Parameter-tuning and assessment by consensus-clustering and silhouette-calculation resulted in seven clusters. Out of these, four contained primarily (>98%) EGF-treated cells, two contained primarily (>94%) CTR cells and one contained a mixture of the two (20.3% CTR, 79.7% EGF). Leiden clusters were sorted according to median placement along PC1 and a heatmap was made of lipid levels within each cluster (Figure 5A). A tSNE-embedding was generated to aid visualization of the clusters (Figures 5B and 5C).

Visual inspection of the heatmap show an overall decrease of multiple lipids in the EGF-majority clusters, consistent with the results from Figure 3, with certain lipids upregulated in clusters C1, C2, C4, and C5 (Figure 5A). Marker lipids were selected based on the following criteria: mean fold change above 1.5 or below 0.667, non-overlapping inter-quartile range (IQR) and statistical significance. All highlighted lipids have *p* < 0.001.

All EGF-majority clusters had reduced levels of PI(32:1), PI(34:1), PI(36:2), and PI(O-34:1) compared to the pooled CTR clusters C6 and C7 (Magenta boxes in Figures 5A and 5D).

Cluster C1 is differentiated from the pooled EGF-clusters by elevated levels of the three measured PG-species. Cluster C2 has elevated levels of PI(36:1) and PI(38:1), as well as low levels of PE(36:2). Cluster C4 has elevated levels of PE(36:2), PE(36:3), PI(34:2) and the highly unsaturated PI(36:3), PI(36:4), PI(38:4), and PI(38:5). Cluster C5 has elevated levels of PA(34:1), PA(36:2), PE(34:2), and the three measured PS-species, as well as reduced levels of PI(38:4) and the three measured PG-species (black boxes in Figure 5A; Figure S2A).

Cluster C3 is the largest EGF-majority cluster. It did not have lipids fulfilling the criteria as markers against the other, pooled EGF-clusters. Using this cluster as baseline for comparison against the pooled CTR-clusters however, we found reduced levels of PA(36:2), PE(36:2), and PE(38:2), multiple mono- and di-unsaturated PI-species, PI(32:0) and PS(36:2). There was additionally increased levels of LPI(18:0) (yellow boxes in Figure 5A; Figure S2B).

The CTR clusters C6 and C7 are differentiated by cluster C6 having reduced levels of PA(36:2), PE(34:2), PE(36:2), PE(36:3), PE(38:2), PI(34:2), and PS(36:2) as well as elevated PG(36:1) (blue boxes in Figure 5A; Figure S2C).

Single-cell analysis showed variable responses to EGF-treatment and clustering analysis revealed distinct lipid profiles. This shows heterogeneous EMT-response at the lipid level.

### EMT induces changes in genes associated with glycerophospholipid biosynthesis

Gene expression was measured by RNA sequencing after 12, 24, 48, and 72 h of EGF-treatment to understand how lipid metabolism is regulated during EMT (see Figures S3A and S3B for clustering and volcano plots of RNA-seq data). Gene set enrichment analysis (GSEA) was performed on significantly differentiated (p.adj < 0.05) genes ranked according to fold change for each EGF-treatment compared to control. The analysis showed significant enrichment for the MSigDB hallmark EMT gene set for each time point (Table S3; Figure S3C), confirming EMT-induction.^42^

**Figure 6:**
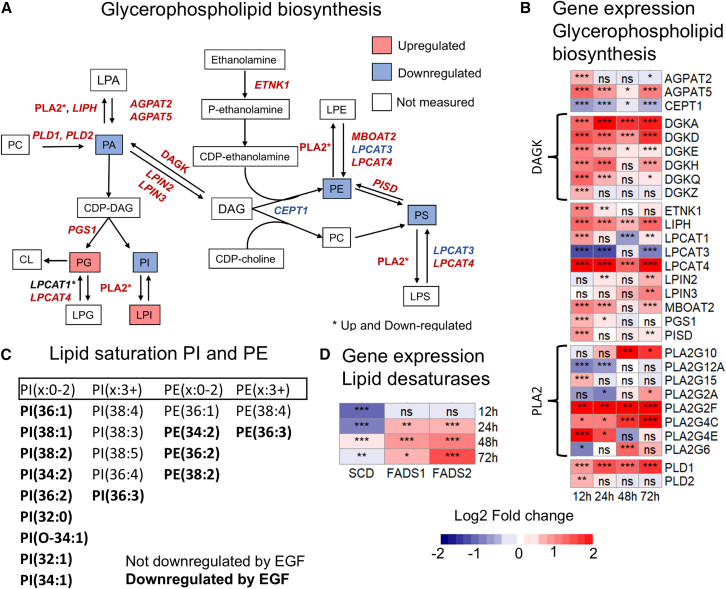
EGF-induced extensive regulation of genes involved in glycerophospholipid biosynthesis in MDA-MB-468 (A) Overview of glycerophospholipid biosynthesis network with metabolites and genes colored red if found to be upregulated and colored blue if found to be downregulated. Network drawn based on the KEGG and Reactome pathway database and the review by Vance.^10^^,^^12^^,^^44^ (B) Overview of up- and downregulated genes in (A) color-coded according to fold change compared to untreated control samples. *N* = 4. (C) Overview of identified PI and PE lipids arranged according to degree of saturation. Significantly downregulated lipids are marked in bold. (D) Overview of up- and downregulated genes involved in lipid desaturation. *N* = 4. *p* values for RNA-seq data in (B and D) were calculated in the SARTools package using a Benjamin-Hochberg-corrected Wald test.^45^ Significance levels: *p* < 0.05: “∗,” *p* < 0.01: “∗∗,” *p* < 0.001: “∗∗∗.”

An equivalent analysis against the glycerophospholipid biosynthesis pathway from the Reactome database did not find significant enrichment for any of the time points; however, the 12 h treatment did display *p* = 0.054.^12^ In order to incorporate fold changes and the lipidomic data, a joint pathway analysis was performed in MetaboAnalyst.^43^ Two lists were supplied, one containing differentially regulated lipids and one containing log2 fold changes of significantly (p.adj < 0.05) regulated genes after 12 h of EGF treatment. Glycerophospholipid metabolism was the most significant hit (*p* = 5.56E−6). An overview of gene expression changes in the glycerophospholipid biosynthesis pathway was constructed using the Reactome database and the review of phospholipid synthesis by Vance (Figure 6A).^10^^,^^12^ Significantly regulated genes with at least 1.5-fold upregulation or 0.667-fold downregulation in at least one measured time point are included in the figure, with gene names colored red if upregulated and blue if downregulated. Genes that are both up- and downregulated are marked with and asterisk. The PLA2 gene family show both up- and downregulated genes but is colored red as we primarily see an upregulation. See Figure 6B for visualization of fold change, significance levels, and timing of gene regulation.

A prominent feature was upregulation of multiple PLA2-genes. PLA2 convert glycerophospholipids to lysophospholipids.^46^ Of note is a persistent upregulation of Lipase H (*LIPH*) which has PA-specific PLA2-activity.^47^ We also found upregulation of 1-acylglycerol-3-phosphate O-acyltransferases 2 and 5 (*AGPAT2* and *5*). The AGPATs are responsible for synthesizing PA from lysophosphatidic acid (LPA).^48^^,^^49^ There was upregulation of phospholipase D1 and 2 (*PLD1* and *2*), which convert PC into PA. PA can be converted to diacylglycerol (DAG) via the activities of Lipin 1-3 (*LPIN1-3*).^50^ *LPIN2* and *3* were upregulated, though did not significantly breach 1.5-fold change until the 72 h measurement. Meanwhile, EGF stimulation lead to a quick upregulation of 6 DAG kinase (DAGK) genes at 12 h, which remained above 1.5-fold change at 24 h for five genes and at 72 h for three genes. DAGK converts DAG back into PA.^51^

DAG is involved in PE, PS, and PC synthesis. Downstream of DAG, there was a 0.66-fold downregulation of choline/ethanolamine phosphotransferase 1 (*CEPT1*) (Figure 6). *CEPT1* combines DAG with CDP-ethanolamine or CDP-choline to make PE or PC, respectively.^52^ PS may be synthesized from either PC or PE. PS may also be converted back to PE by phosphatidylserine decarboxylase (*PISD*), which had a 1.82-fold upregulation at 12 h.^53^ PE and PS can be de-acylated to LPE and LPS, respectively, by PLA2, which was primarily upregulated.^46^ There was also both up- and downregulation of genes responsible for the inverse reaction.^54^^,^^55^^,^^56^^,^^57^^,^^58^^,^^59^

CDP-DAG is used for PI and PG-synthesis. Downstream of CDP-DAG, there was a 1.54-fold upregulation of phosphatidylglycerophosphate synthase 1 (*PGS1*)*,* which is considered rate-limiting in PG-synthesis (Figure 6).^60^^,^^61^ PG and PI can both be de-acylated by PLA2.^46^ There was an initial 1.97-fold increase in lysophosphatidylcholine acyltransferase 1 (*LPCAT1*) at 12 h, as well as a 0.64-fold downregulation at 48 h*.* Meanwhile *LPCAT4* had an initial 6.6-fold upregulation at 12 h and was persistently upregulated for all time points. *LPCAT4* and *LPCAT1* encode proteins that convert lysophosphatidylglycerol (LPG) into PG.^55^^,^^62^^,^^63^^,^^64^

In addition to headgroups, glycerophospholipids can be categorized according to the saturation of their fatty acid tail. Nine PI species with two or fewer double bonds were identified, and all of these were significantly reduced in the EGF-treatment compared to CTR (Figures 3 and 6C). Out of the identified PI species with more than two double bonds, only one was significantly downregulated by the EGF treatment (Figure 6C). A similar trend was observed among the PE species, with three out of four low-saturation and one out of two high-saturation lipids affected by EGF. The fatty acid saturation can be adjusted by PLA2 and LPLATs interconversion between glycerophospholipids and lysophospholipids.^24^ For LPLATs to reduce glycerophospholipid saturation, they need unsaturated fatty acids, so we checked the expression of fatty acid desaturases. Stearoyl-CoA desaturase (*SCD*), which converts saturated (x:0) fatty acids to mono-unsaturated (x:1) fatty acids were downregulated.^65^ Meanwhile, fatty acid desaturase 1 and 2 (*FADS1* and *FADS2*), which are involved in making poly-unsaturated fatty acids were both upregulated by EGF (Figure 6D).^66^^,^^67^ This indicates a shift away from glycerophospholipids with low saturation.

To assess whether the observed lipid regulation can be found in other EMT contexts, we used single-cell gene expression data generated by Cook and Vanderhyden.^68^ The expression of glycerophospholipid biosynthesis genes as well as fatty acid desaturases were analyzed in four different cell lines exposed to three different EMT-inducers (Section S5). Direct comparisons of regulation after 1 and 3 days, respectively, of EMT-induction showed little regulation for the specific time points, with the most notable being membrane bound glycerophospholipid O-acyltransferase 2 (*MBOAT2*) which was significantly upregulated in four conditions (Figure S4). A pseudotime analysis was performed to get an overview of trends using the full dataset (Figure S5). Consistent with our results, *AGPAT2* was upregulated in seven and downregulated in two of the EMT-inductions. Similar results were found for the *MBOAT*s, with a majority of inductions having upregulation of either *MBOAT2* or *MBOAT7*. Inconsistent with our results, *PLA2G12A* was downregulated in four and upregulated in one EMT-induction, while *FADS1* had divergent response as it was downregulated in four and upregulated in three inductions.

Gene expression data indicate specific regulation of glycerophospholipid biosynthesis and saturation. Follow-up analysis of gene expression in multiple EMT-contexts shows regulatory changes both consistent and inconsistent with our results.

## Discussion

In this study, we investigated how EMT affects lipid metabolism and explored lipid-level heterogeneity in MDA-MB-468 breast cancer cells. EMT heterogeneity is well documented at the gene expression level; however, there has been little focus on the lipid level. EMT was found to cause an overall reduction in 2 PA, 2 PS, 4 PE, and 11 PI-species, as well as increase in two PG species and LPI(18:0) (Figure 3). This was accompanied by regulation of genes involved in glycerophospholipid biosynthesis (Figures 6A and 6B). EMT caused cells to disperse, and dispersed cells were more EMT-like than cohesive cells (Figures 2D and 4). Heterogeneity of lipid-regulation goes beyond the cohesive vs. dispersed divide, however, as single cell analysis revealed multiple clusters with differing lipid levels (Figure 5).

MALDI-MSI measurements indicated significant changes in glycerophospholipid metabolism following EGF-induced EMT. Gene expression analysis confirmed altered regulation of several metabolic reactions involving these phospholipids. PA is a signaling lipid which also acts as a precursor for downstream glycerophospholipid biosynthesis. PA activates pro-metastatic signaling cascades, yet its levels were reduced despite upregulation of PA-synthesizing *PLD1/2* and DAGK (Figures 6A and 6B).^19^ PA is converted to DAG by upregulated *LPIN2/3*, which may have counteracted some of the DAGK activity. DAGK is activated by the phosphoinositide pathway, which promotes cancer progression, and *DGKA* has been found to promote metastasis, migration, and invasion.^16^^,^^69^^,^^70^^,^^71^^,^^72^^,^^73^^,^^74^

Downstream of DAG, *CEPT1* combines DAG with CDP-ethanolamine to make mono- and di-unsaturated PE in the final step of the CDP-ethanolamine pathway.^75^ *CEPT1* was downregulated, which is consistent with the observed reduction in PE-levels (Figure 6A). PE(16:1/18:0 and 16:0/18:1) and PE(18:0/18:2 and 18:1/18:1) has been found to be significantly lower in metastatic MDA-MB-231 breast cancer cells than non-metastatic T-47D breast cancer cells.^20^ Additionally, the CDP-ethanolamine pathway has been found to inhibit mesenchymal genes in mouse embryonic fibroblasts.^76^

PS-levels were reduced, and PS signaling is involved in removing apoptotic bodies. This activity is, however, regulated by PS relocating to the outer plasma membrane, rather than by adjusting overall cellular levels. The observed reduction was unexpected as other studies have found either no difference or higher PS-levels in metastatic cell lines.^20^ For instance, Kim et al. found the levels of multiple PS-species to be higher in metastatic MDA-MB-231 than non-metastatic MCF-7, including PS(34:1) and PS(36:2).^21^ Our observed upregulation of *PISD* suggests that the reduction in PS-levels are at least partially due to it being converted to PE.

In addition to acting as a precursor to PE and PS via DAG, PA can be converted to PG and PI via CDP-DAG. We observed an upregulation of *PGS1*, which is considered rate-limiting in PG-synthesis, together with increasing levels of PG-species (Figures 6A and 6B). This could indicate that PA decreases due to increased conversion into PG; however, a large fraction of EGF-treated cells had PG-levels remain stable while PA decreased (Figure 5A). PA thus appears to decrease independent of the increase in PG. PG primarily acts as a precursor to cardiolipin, an important constituent of mitochondrial membranes. Mitochondria are known to alter their activity to promote metastasis, and cardiolipin is essential to maintain mitochondrial function.^77^^,^^78^ We did not find significant changes in *CRLS1*, which synthesizes cardiolipin from PG. Further studies are needed to determine the effect of this PG-increase on cardiolipin and, further, on mitochondrial function. In addition to its role in cardiolipin synthesis, PG is an activator of protein kinase C, which in turn can promote EMT.^79^^,^^80^

Also, downstream of CDP-DAG were decreasing PI-levels accompanied by increasing LPI. LPI has been found to have a pro-metastatic function in breast cancer cells, where it activates GPR55 to promote migration and elongation.^81^ LPI is made by the action of PLA2 family members. PLA2 hydrolyzes glycerophospholipids at the sn2 position to make a lysophospholipid and a fatty acid. They have long been considered important in cancer and as potential therapeutic targets.^82^ PLA2 family members have different specificities not fully delineated.^46^ We found different PLA2-genes to be up- and/or downregulated at different time points, indicating a fine-tuning of their action which resulted in higher levels of LPI.

Since LPI(18:0) was the only detected lysophospholipid we cannot determine to what extent altered PLA2-activity affects the hydrolyzation for the other headgroups. However, we note the 2.34-fold upregulation of PA-specific *LIPH*, which de-acylates PA into LPA. LPA is a pro-tumorigenic lysophospholipid that has been found to promote migration and identified as an EMT-inducer.^83^^,^^84^^,^^85^ Increased shuffling of PA toward LPA would further explain decreasing PA-levels despite increased DAGK and *PLD1/2*. Upregulation of two *AGPAT*-species adds further ambiguity regarding the balance between PA and LPA however. Decreasing PA-levels were accompanied by decreasing levels of PE and PS. In addition to the mentioned regulation of genes involved in their biosynthesis, both lipids may be affected by PLA2-activity. There was, however, diverging regulation of genes responsible for converting PE and PS to LPE and LPS (Figures 6A and 6B). There may have been an adjustment of lipid saturation, however, as this is regulated during conversion between glycerophospholipids and lysophospholipids.^24^

An interesting finding was that the majority of changes in lipid composition following EGF-stimulation could also be found in dispersed CTR cells. This is consistent with the assumption that phenotypical and biochemical changes are strongly associated. EMT is associated with changes in cellular traits such as cell-cell adhesivity and motility, affecting the overall state of the cell population. These traits are affected by EMT transcription factors which are also known to regulate metabolism.^86^ Using the inherent spatial resolution of MALDI-MSI allows metabolic profiling of selected sub-populations of cells, thereby providing a more detailed insight into the associations between microscopic phenotype and cellular biochemistry. The method is limited by its sensitivity, as we were unable to detect low-abundance lipids. Higher lateral resolution would have allowed confident separation and single-cell analysis of individual cohesive cells in addition to the dispersed ones. Our results corroborate the involvement of phospholipid metabolism in the metastatic process in cancer, but further research is required for functional understanding of these processes and whether specific manipulation of phospholipid metabolism can inhibit EMT in cancer cells.

The levels of multiple glycerophospholipids are reduced and LPI(18:0) as well as two PG-species increase during EGF-induced EMT in MDA-MB-468 breast cancer cells. There is overlap between changes in lipid-levels during EMT and changes observed in untreated cells that disperse. Single-cell analysis show heterogeneous EMT-response at the lipid-level. Gene expression analysis show extensive regulation of genes involved in glycerophospholipid biosynthesis. This demonstrates that EMT causes altered gene expression resulting in altered phenotype at the lipid-level.

### Limitations of the study

A limitation of this study is that more highly saturated lipids were measured than highly unsaturated species. For instance, none of the measured PA-, PS-, and PG-species had more than two double bonds in their two hydrocarbon chains. Unsaturated lipids promote membrane fluidity and in turn cell migration, making lipid saturation an important property of EMT.^22^^,^^23^ If we look at the PI-species, nine out of nine low-saturated lipids (PI(x:0–2)) were reduced in the EGF-treatment, compared to one out of five highly unsaturated lipids (PI(x:3+)) (Figure 6C). Studies capturing a larger span of fatty acid chains could help determine whether our observed decrease in PI-, PE-, PA-, and PS-species represent changes in membrane head group composition or simply a shift in saturation levels.

Besides the aforementioned limited number of detected lipids, it must be acknowledged that the study is limited to lipidomic data from a single EMT-inducer in a single-cell line. Future studies should consider lipid-level changes during EMT and cancer progression in multiple model systems and, ideally, patient samples. Another limitation is that the MALDI-MSI method does not identify apoptotic and mitotic cells. Integrating information regarding cell state would aid interpretation of the observed lipid-level changes.

## Resource availability

### Lead contact

Requests for further information and resources should be directed to and will be fulfilled by the lead contact, Ellen Marie Botne Quinsgaard (embquins@gmail.com).

### Materials availability

This study did not generate new unique reagents.

### Data and code availability

#### Data

• Raw metabolite data have been made available at the MetaboLights database (https://www.ebi.ac.uk/metabolights/MTBLS12650/) with the study identifier MTBLS12650.
• Raw and processed bulk RNAseq data are available from the NCBI Gene Expression Omnibus under accession code GSE325402.
• Raw single-cell RNAseq data of EMT-induction in cell lines published by Cook and Vanderhyden^68^ are available from the NCBI Gene Expression Omnibus under accession code GSE147405.
• Processed metabolite data are available from Mendeley data at https://doi.org/10.17632/4t5g63m65h.1.
• Data reported in this paper will be shared by the lead contact upon request.

#### Code

This paper does not report original code.

#### Other items

• An analysis report on the bulk RNAseq data is available from Mendeley data at https://doi.org/10.17632/4t5g63m65h.1.
• Any additional information required to reanalyze the data reported in this paper are available from the lead contact upon request.

## Acknowledgments

RNA sequencing and purity assessment, as well as bioinformatics analysis with transcriptomic mapping, count matrix generation, gene normalization, and differential analysis was performed by the Genomics Core Facility, Norwegian University of Science and Technology (NTNU). The MALDI MSI analysis and sample preparation was performed at the MR Core Facility, Norwegian University of Science and Technology (NTNU). MALDI FT-ICR MS analysis for lipid identification was performed at SINTEF Industry. The Genomics and MR core facilities are funded by the Faculty of Medicine and Health Sciences at 10.13039/100009123NTNU and Central Norway Regional Health Authority. This project was supported by grants from the Liaison Committee for Education, Research and Innovation in Central Norway (project ID 90434400) and the Norwegian cancer society (project ID 198069). This study was also supported by Kreftfondet, 10.13039/501100011769St. Olavs hospital (grant no. 36-22).

## Author contributions

E.M.B.Q., conceptualization, data curation, formal analysis, investigation, methodology, validation, visualization, writing – original draft, and writing – review and editing; M.G., conceptualization, data curation, formal analysis, investigation, methodology, visualization, and writing – review and editing. V.W.A., formal analysis, investigation, and methodology; S.K., formal analysis, methodology, and writing – review and editing; A.N., investigation and writing – review and editing; J.I., formal analysis; J.V.S., formal analysis and writing – review and editing; S.A.M., conceptualization, funding acquisition, project administration, and writing – review and editing.

## Declaration of interests

The authors declare no competing interests.

## STAR★Methods

### Key resources table

**Table:** 

REAGENT or RESOURCE	SOURCE	IDENTIFIER
**Chemicals, peptides, and recombinant proteins**		

Epidermal Growth Factor (EGF)	Peprotech	Cat#: AF-100-15
Buffered formaldehyde	VWR Chemicals	Cat# 9713.1000
Ammonium acetate	ThermoFisher Scientific	Cat# R1181
*N*-(1-Naphthyl)ethylenediamine dihydrochloride (NEDC)	Sigma-Aldrich	N9125-100G

**Deposited data**		

Raw MALDI data	This paper; MetaboLights	https://www.ebi.ac.uk/metabolights/MTBLS12650/
Processed MALDI data	This paper; Mendeley Data	https://doi.org/10.17632/4t5g63m65h.1
Raw and Processed Bulk RNAseq data	This paper; NCBI GEO	GSE325402
Single-cell RNAseq data	Cook and Vanderhyden^68^	https://doi.org/10.1038/s41467-020-16066-2; GEO: GSE147405

**Experimental models: Cell lines**		

MDA-MB-468	ATCC	Cat# HTB-132; RRID:CVCL_0419

**Oligonucleotides**		

Primers for *SNAI1*,*TWIST1, VIM, EZR, FN* and *GAPDH,* see Table S1	Genosys	Cat# KSPQ12012G

**Software and algorithms**		

FlexImaging	Bruker Daltonics	https://www.bruker.com/en/services/software-downloads.html
R	R core team^87^	https://www.r-project.org/
ScilsLab	Bruker Daltonics	https://www.bruker.com/en/products-and-solutions/mass-spectrometry/ms-software/scils-lab.html
Python	Python development team	https://www.python.org/
ImageJ	Schneider et al.^88^	https://imagej.net/ij/

### Experimental model and study participant details

#### Cell line

The human breast cancer cell line MDA-MB-468 (RRID:CVCL_0419) was purchased and used with permission from ATCC. The cells were neither authenticated nor tested for mycoplasma contamination after delivery. The line was maintained in DMEM:F12 cell medium with HEPES (Gibco, 3133038) supplemented with 5% Fetal Bovine Serum (FBS)(Gibco, 10270) and 50 U/mL Penicillin-Streptomycin (P/S) (Gibco, 15070063). Cells were cultured in an incubator at 37°C and 5% CO_2_.

### Method details

#### EMT induction

Medium was replaced with FBS-free culture medium 24 hours after seeding. After another 24 hours in FBS-free medium cells were stimulated with epidermal growth factor (EGF) (Peprotech, AF-100-15). MDA-MB-468 has elevated levels of epidermal growth factor receptor (EGFR) and EGF is an established inducer of EMT in this cell line.^89^^,^^90^^,^^91^

#### qPCR

Cells were seeded out in 6-well culture plates, 200 000 cells per well in 2 mL media per well. Cells were treated with 25 ng/mL EGF for, respectively, 0, 12, 24, 48 or 72 hours. Three wells were seeded for each experimental condition. RNA was extracted using TRI Reagent Solution (Invitrogen, AM9738). All samples were seeded and harvested together, with the only differentiating factor being time of EGF-stimulation. cDNA was synthesized using High Capacity RNA-to-cDNA kit (Applied Biosystems, 4387406) according to the manufacturer’s protocol. Quantitative PCR was performed using Perfecta SYBR Green Fastmix Rox (QuantaBio, 95073) on a StepOnePlus Real-time PCR System (Applied Biosystems). Primers for *SNAI1*, *TWIST1*, *VIM*, *EZR*, *FN1* and the reference gene *GAPDH* were used for gene amplification (Genosys, KSPQ12012G). Primer sequences can be found in Table S1. Relative RNA-expression was calculated using the delta delta Ct-method.

#### MALDI MSI slide preparation

Indium Tin Oxide (ITO)-slides (Bruker Daltonics GmbH, 8237001) were placed in 10cm cell culture dishes, sterilized in 75% ethanol and rinsed with sterile Phosphate Buffered Saline (PBS, Sigma-Aldrich, D8537). 500.000 cells in 10mL medium were seeded out per culture dish. Cells were stimulated with 25ng/mL EGF for 72 hours. Morphology was visually assessed to verify EMT-induction. Medium was removed from the slides and they were rinsed with PBS. Slides were fixed in 4% buffered formaldehyde (pH 7, stabilized in 0.5-1.5% methanol) (VWR Chemicals, 9713.1000) for 2 minutes, then rinsed once with PBS and twice with 150 mM ammonium acetate (Thermofisher Scientific, R1181). Each slide was then air-dried under a N_2_-stream. Afterwards the slides were covered with the MALDI matrix *N*-(1-Naphthyl)ethylenediamine dihydrochloride (NEDC, Sigma-Aldrich, N9125-100G, 7 g/L in 70% methanol) using an M5 HTX sprayer (HTX technologies, LLC) with the following parameters: number of passes 18, flow rate of 0.06 mL/min, velocity of 1200 mm/min, track spacing 3 mm, N_2_ flow 2 L/min, spray pattern CrissCross, nozzle height 40 mm and N_2_ pressure of 10 psi. The resulting matrix density was 2.1 mg/mm^2^. To control for technical variations in MALDI-MSI, the samples were run on three separate days, with three replicates of each treatment on each day. Additionally, control (CTR) and EGF-samples were paired up to share matrix application runs.

#### MALDI MSI of metastatic breast cancer cells

MALDI MSI measurements were performed using a rapifleX MALDI Tissuetyper™ time-of-flight mass spectrometer (Bruker Daltonics, Bremen, Germany). The analyzer was operated in negative reflector mode, and the laser was fired with a repetition rate of 10 kHz using a lateral resolution of 10 μm and 300 shots per pixel. External calibration was performed using red phosphorus dispersed in acetone (100 mg/mL), spotted on a cell-and matrix-free area. Mass spectra were acquired in the m/z range 500–1000 with a digitizer frequency of 1.25 GHz.

#### Lipid identification by high mass resolution MALDI FT-ICR MS

Accurate mass was acquired using a 12T Bruker Fourier-transform ion cyclotron resonance (FT-ICR) mass spectrometer (Solarix, Bruker Daltonics, Bremen, Germany) equipped with a dual MALDI/ESI ion source. Samples were measured using a spatial resolution of 20 μm and the minimum laser spot size. A total of 20 laser shots were used per pixel. Data was collected in negative ionization mode in the mass range m/z 150–1300. External calibration was performed prior to analysis using the electrospray ion source and NaTFA clusters. FlexImaging (Bruker Daltonics) was used to visualize the imaging data and Bruker DataAnalysis was used to visualize individual mass spectra. After identifying cell specific peaks of interest, those peaks were examined manually in the high mass resolution MSI dataset acquired by MALDI FT-ICR. The found m/z values were compared with a homebuilt lipidomics database provided by Lipometrix (KU Leuven) and putatively identified considering a mass error < 2 ppm (See Table S2).

#### Identification of dispersed single cells and cohesive groups of cells

Two brightfield images were taken of each sample. Brightfield images were analyzed to categorize cell areas as containing either one single dispersed cell or multiple cohesive cells. Brightfield pixels containing cells were identified using gaussian blurring with a standard deviation of 5 px in X and Y direction followed by binary thresholding using Otsu’s method to generate a binary mask as input for contour detection using the findContours function as implemented in the OpenCV package for Python.^92^^,^^93^ Contours were initially filtered by size (100-2500 μm^2^) and aspect ratio (between 0.75 and 1.25) of their rectangular bounding box followed by manual inspection and adjustment to obtain only areas containing single cells. MSI and brightfield images were combined using imageJ to assign single cell IDs to MSI pixels.^88^ The assignment of cell-containing MSI pixels to bright-field IDs were adjusted in R using a combination of EBimage’s Floodfill function before a final, manual adjustment of pixel assignment and categorization of dispersed vs cohesive cell areas.^94^

#### RNA-sequencing

Cells were seeded out in 6-well culture plates, 200 000 cells per well in 2 mL media per well. Untreated controls were compared to cells treated with 50 ng/mL EGF for 12, 24, 48 and 72 hours respectively, with four biological replicates per treatment group. RNA was extracted using the RNeasy Mini kit (Qiagen, 74104) according to the manufacturer’s instructions. RNA quantity, purity, and integrity were measured on an Agilent 2100 Bioanalyzer. Library preparation was performed using the Lexogen Corall mRNA protocol, and sequencing was done using the Illumina NS500 HO flow cell at 75 bp SE reads.

### Quantification and statistical analysis

Statistical analyses were performed in R.^87^

#### qPCR

After calculating relative gene expression using the delta delta Ct-method, significance levels were calculated using pairwise t tests of each treatment group vs CTR. P-values were adjusted using the Bonferroni-method.

##### Identification of cell area and peak picking of MSI datasets

One MSI image was taken of each sample. MSI datasets were converted to imzML in FlexImaging (Bruker Daltonics) and pre-processed using the Cardinal package for R.^95^ TIC normalization, peak-picking with threshold of signal to noise ratio of 6 and peak-alignment were applied to obtain a centroided processed dataset. Spatial K mean clustering was used to identify cell specific clusters and subset the datasets. MSI images were generated in ScilsLab (Bruker Daltonics, Bremen, Germany). Mean spectra were extracted from areas containing cells and areas without cells. Cell specific peaks were identified by filtering for peaks with minimum 5 times stronger signal in cell spectra than in cell-free spectra. The resulting peaks were used for further analyses.

#### Comparison of cell-pixel numbers

The percentage of cell-containing pixels between treatment groups, and the percentage of dispersed cell-containing pixels were both compared using two-sample t tests.

#### Processing and analysis of MALDI-MSI data

To compare the overall changes in lipid levels between treatment and control groups, a mean spectrum was extracted from each sample based on all pixels found to contain cells. A paired t test was used to identify lipid peaks that differed significantly between treatment groups. Samples were paired according to rounds of matrix application. A Bonferroni-correction was used to account for multiple testing.

To compare dispersed vs cohesive cells we pooled all pixels from the two types of cell organization, respectively, in each sample. Mean spectra from the two categories of cell spectra were extracted and assessed using a PCA-analysis on scaled data. Differences between cohesive and dispersed cells within each treatment group were quantified using a paired t test with spectra paired according to which sample they were extracted from. A Bonferroni-correction was applied.

For single-cell analyses one mean spectrum was extracted from each collection of pixels found to belong to a single dispersed cell. That is, multiple spectra from each sample. A batch-correction was performed on the single-cell data based on which samples had been paired up during matrix application. Batch-correction was done in R using Combat from the sva-package.^87^^,^^96^^,^^97^ Data was scaled before clustering.

A shared nearest neighbor (SNN)-graph was constructed using the scran-package for R.^98^^,^^99^ Clustering of the SNN-graph was performed using the Leiden algorithm.^100^ To find the ideal set of clusters consensus clustering was performed in the following way: Data was randomly sampled using a resampling rate of 0.8 in 500 iterations. For each iteration, combinations of SNN-graphs with Leiden clusters were constructed using a set of eight values for the number of nearest neighbors and 19 values for resolution. Resulting cluster assignments were used as input for constructing consensus matrices with differing number of clusters. From each of these consensus matrices a set of cluster assignments were extracted using hierarchical clustering with McQuitty distances. The final optimal number and set of clusters were determined based on consensus heatmaps and silhouette calculation on a PCA-embedding. The optimal number of clusters was determined to be 2, followed by 7. Since we were interested in exploring heterogeneity beyond CTR vs EGF, we chose to go along with 7 clusters. A PCA-analysis was performed on scaled, batch-corrected data and colored according to treatment and Leiden clusters respectively. Leiden clusters were named and sorted according to median placement along PC1. A heatmap was made of scaled data using the pheatmap-package for R to visualize lipid levels in the Leiden clusters.^101^ A t-SNE-embedding of scaled data was constructed to further aid cluster visualization.

Marker lipids were identified to describe the clusters. Three requirements were set for a lipid to be considered a marker between a pair of clusters: Fold change above 1.5 or below 0.667, significant p-value as determined by the two-sample t test (p.adj<0.01) and non-overlapping interquartile range (IQR). P-values were adjusted using the Bonferroni approach. Marker lipids were identified to determine what separated the two CTR-majority clusters, what differentiated each EGF-majority cluster from the rest of the EGF-majority clusters and which lipids differentiated every EGF-majority cluster from the two CTR-majority clusters. When comparing individual clusters against a set of clusters, the latter were pooled together as a single group with each datapoint representing a cell, rather than a cluster. I.e. larger clusters were weighed more strongly than smaller clusters.

#### Analysis of RNA sequencing data

Gene normalization and differential analysis was performed using the DESeq2 and Bioconductor packages in R according to the SARTools pipeline.^45^^,^^87^^,^^102^^,^^103^^,^^104^ P-values were calculated using the Wald test and a BH p-value adjustment was performed to account for multiple testing with the level of controlled false positive rate set to 0.05.^105^

#### Pathway and enrichment analyses

Gene set enrichment analysis was performed using the fgsea-package in R against the MSigDB hallmark EMT and Reactome 2024 Glycerophospholipid Biosynthesis gene sets.^12^^,^^42^^,^^87^^,^^106^ Enrichment was performed for each EGF-treatment compared to the control, with input being significantly (p.adj<0.05) differentiated genes ranked according to fold change.

A joint pathway analysis was conducted using MetaboAnalyst 6.0.^43^ Input was log2 fold changes of genes with significant (p.adj<0.05) regulation after 12 hours of EGF-treatment, as well as a list of lipids that were significantly (p.adj<0.05) regulated in the EGF vs CTR treatments. The analysis was performed using the integrated metabolic pathways database with enrichment analysis using the hypergeometric test, degree centrality as topology measure and unweighted p value combination for the integration method.

Heatmaps of genes in the glycerophospholipid biosynthesis pathway were generated using the pheatmap package for R.^101^ Input data was log2 Fold changes of each EGF-treatment compared to control. Genes were included in the heatmap if they had significant (p.adj<0.05) differential expression and fold change above 1.5 or below 0.667 in at least one of the EGF-treatments compared to control.

#### Analysis of single-cell RNAseq data

Pre-processed, pseudo temporally ordered single-cell RNA-seq data was acquired from Cook and Vanderhyden.^68^ Mean fold change after 1 and 3 days, respectively, of EMT-induction was calculated and significance levels were determined using the Wilcoxon rank sum test. Holm p-value adjustments were performed, and genes significantly (p.adj<0.05) regulated with a fold change above 1.5 or below 0.667 were counted. Counts of gene regulations were organized in a table together with bulk RNAseq data for the relevant timepoints and genes.

Pseudotime coefficients were calculated for genes in the glycerophospholipid biosynthesis pathway using the gam function from the mgcv package for R using the model exp∼pseudotime + batch. The gene list was acquired from the Reactome database.^12^ P-values were adjusted using the Benjamin-Hochberg false discovery rate and significant genes (p.adj <0.05) were visualized using a heatmap.
