# Supplementary material for: Single-cell lipidomic analysis of the epithelial-mesenchymal transition using mass spectrometry imaging
Source: iScience. 2026 Apr 21;29(6):115847. doi: 10.1016/j.isci.2026.115847 (PMC13156679; doi:10.1016/j.isci.2026.115847)
Supplement: Document S1. Figures S1–S5 and Tables S1–S3 [file mmc1.pdf]

iScience, Volume *29*

Supplemental information

Single-cell lipidomic analysis of the epithelial-mesenchymal transition using mass spectrometry imaging

Ellen Marie Botne Quinsgaard, Marco Giampà, Veronica With Andreassen, Sebastian Krossa, Anna Nordborg, Jakub Idkowiak, Johannes V. Swinnen, and Siver Andreas Moestue

Supplementary

For additional code and datafiles generated by this study see Mendeley Data (https://data.mendeley.com), doi: 10.17632/4t5g63m65h.1, Metabolights (https://ebi.ac.uk/metabolights) under study identifier MTBLS12650 and the NCBI Gene expression Omnibus under study identifier GSE325402.

1. Primer sequences qPCR

Table S1 Primers used during qPCR. Related to STAR Methods. Purchased from Genosys (KSPQ12012G)

Gene	Oligo name	Sequence
SNAI1	FH1_SNAI1	5'-CTCTAATCCAGAGTTTACCTTC-3'
SNAI1	RH1_SNAI1	5'-GACAGAGTCCCAGATGAG-3'
TWIST1	FH1_TWIST1	5'-CTAGATGTCATTGTTTCCAGAG-3'
TWIST1	RH1_TWIST1	5'-CCCTGTTTCTTTGAATTTGG-3'
Vimentin	FH1_VIM	5'-GGAAACTAATCTGGATTCACTC-3'
Vimentin	RH1_VIM	5'-CATCTCTAGTTTCAACCGTC-3'
Fibronectin	BH1_FN1	5'-CAAGTACAATCTACCATCATCC-3'
Fibronectin	FH1_FN1	5'-CCATAGCTGAGAAGTGTTTTG-3'
Ezrin	BH1_EZR	5'-CTCCTTTTCTTCTCTGTTTCC-3'
Ezrin	FH1_EZR	5'-TGACTTTGTGTTTTATGCCC-3'
RPS13	BH1_RPS13	5'-CATATTTCCAATTGGGAGGG-3'
RPS13	FH1_RPS13	5'-GAAAGCATCTTGAGAGGAAC-3'

2. Lipid identification

Table S2: Overview of lipid identification of peaks of interest. Related to STAR methods. Table only lists peaks with min 5 times stronger signal in cell-spectra than in cell-free spectra. Below are measured mz by FT-ICR, theoretical mz according to the Lipometrix database, ion of the identified lipid, lipid annotation and whether we consider the mz signal to be identified.

FT-ICR mz	Theoretical mz	Ion	error	Annotation	Identified
599.3202	599.3202		0.010011	LPI 18:0 [M-H]	TRUE
673.4809	673.4814		-0.67708	PA 34:1 [M-H]	TRUE
687.5443	687.4031		205.4733	PA 36:8 [M-H]	FALSE
699.4967	699.497		-0.4589	PA 36:2 [M-H]	TRUE
701.5155	701.5127	[M-H]-	4.008481	PA 36:1	FALSE
714.5078	714.5079	[M-H]-	-0.14416	PE 34:2	TRUE
716.5271	716.5236	[M-H]-	4.872135	PE 34:1	FALSE
740.5233	740.5236		-0.41862	PE 36:3 [M-H]	TRUE
742.5393	742.5392	[M-H]-	0.075417	PE 36:2	TRUE
743.5422	743.5596	[M-H]-	-23.4069	isotope	FALSE
744.5544	744.5549	[M-H]-	-0.62991	PE 36:1	TRUE
745.5583	745.5753	[M-H]-	-22.832	isotope	FALSE

747.5176	747.5182	[M-H]-	-0.84279	PG 34:1	TRUE
748.5213	748.5287	[M-H]-	-9.83817	isotope	FALSE
760.5129	760.5134		-0.61537	PS 34:1 [M-H]	TRUE
766.5388	766.5392		-0.47356	PE 38:4 [M-H]	TRUE
770.57	770.5705		-0.70986	PE 38:2 [M-H]	TRUE
773.5332	773.5338		-0.78342	PG 36:2 [M-H]	TRUE
775.5491	775.5495	[M-H]-	-0.56734	PG 36:1	TRUE
786.5291	786.5291	[M-H]-	0.021614	PS 36:2	TRUE
788.5439	788.5447	[M-H]-	-1.01072	PS 36:1 [M-H]	TRUE
790.5513	790.5604		-11.5399	PS 36:0 [M-H]	FALSE
795.5503	795.5182		40.3938	PG 38:5 [M-H]	FALSE
797.6535	797.5127		176.6016	PA 44:9 [M-H]	FALSE
799.6692	799.5495		149.6493	PG 38:3 [M-H]	FALSE
807.5025	807.5029	[M-H]-	-0.5548	PI 32:1	TRUE
808.506	808.5134	[M-H]-	-9.19046	isotope	FALSE
809.5184	809.5186		-0.2483	PI 32:0 [M-H]	TRUE
821.5549	821.5549	[M-H]-	0.001217	PI O-34:1	TRUE
833.5178	833.5186	[M-H]-	-0.93819	PI 34:2	TRUE
834.5208	834.5291	[M-H]-	-9.92431	isotope	FALSE
835.5331	835.5342	[M-H]-	-1.33208	PI 34:1	TRUE
836.537	836.5447	[M-H]-	-9.22288	isotope	FALSE
837.5403	837.5499	[M-H]-	-11.4668	PI 34:0	FALSE
857.5177	857.5186		-1.00406	PI 36:4 [M-H]	TRUE
859.5331	859.5342		-1.2914	PI 36:3 [M-H]	TRUE
861.5487	861.5499	[M-H]-	-1.43346	PI 36:2	TRUE
862.5526	862.5604	[M-H]-	-9.00813	isotope	FALSE
863.5646	863.5655	[M-H]-	-1.09083	PI 36:1	TRUE
864.5678	864.576	[M-H]-	-9.44231	isotope	FALSE
883.5328	883.5342		-1.54946	PI 38:5 [M-H]	TRUE
886.5524	886.5604	[M-H]-	-9.0578	isotope	FALSE
885.5492	885.5499	[M-H]-	-0.79724	PI 38:4	TRUE
887.5646	887.5655	[M-H]-	-0.98246	PI 38:3	TRUE
889.5802	889.5812		-1.11176	PI 38:2 [M-H]	TRUE
891.5962	891.5968		-0.66173	PI 38:1 [M-H]	TRUE

3. Single-cell clustering

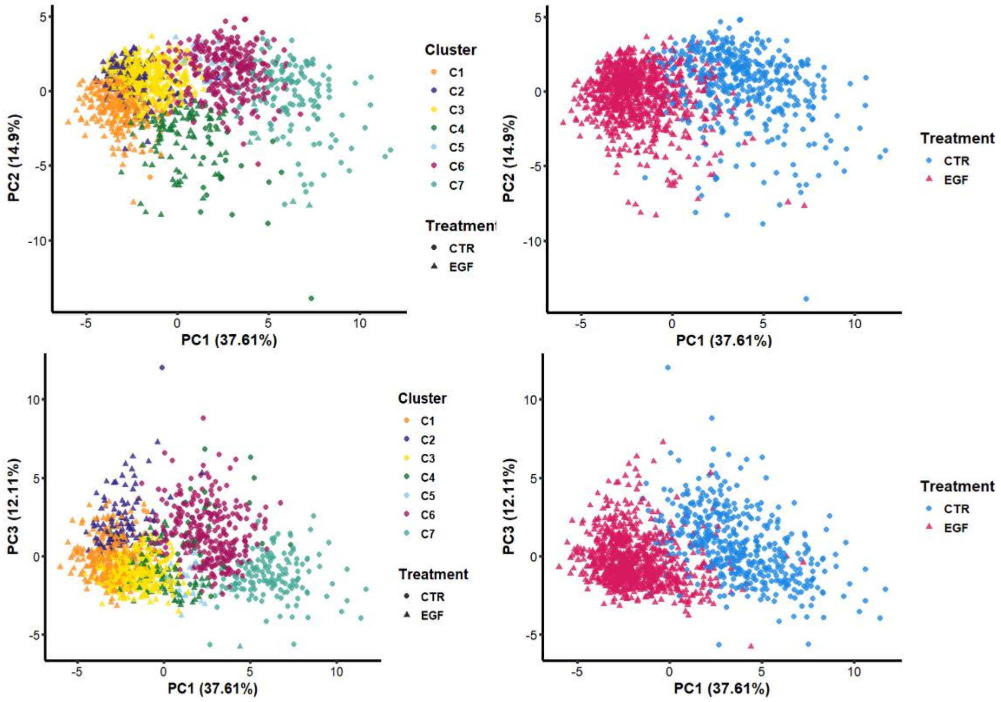

Figure S1: PCA-embedding of single-cell lipid data. Related to Figure 5. Colored according to Leiden clusters and treatment group respectively. Top: PC1 vs PC2. Bottom: PC1 vs PC3.

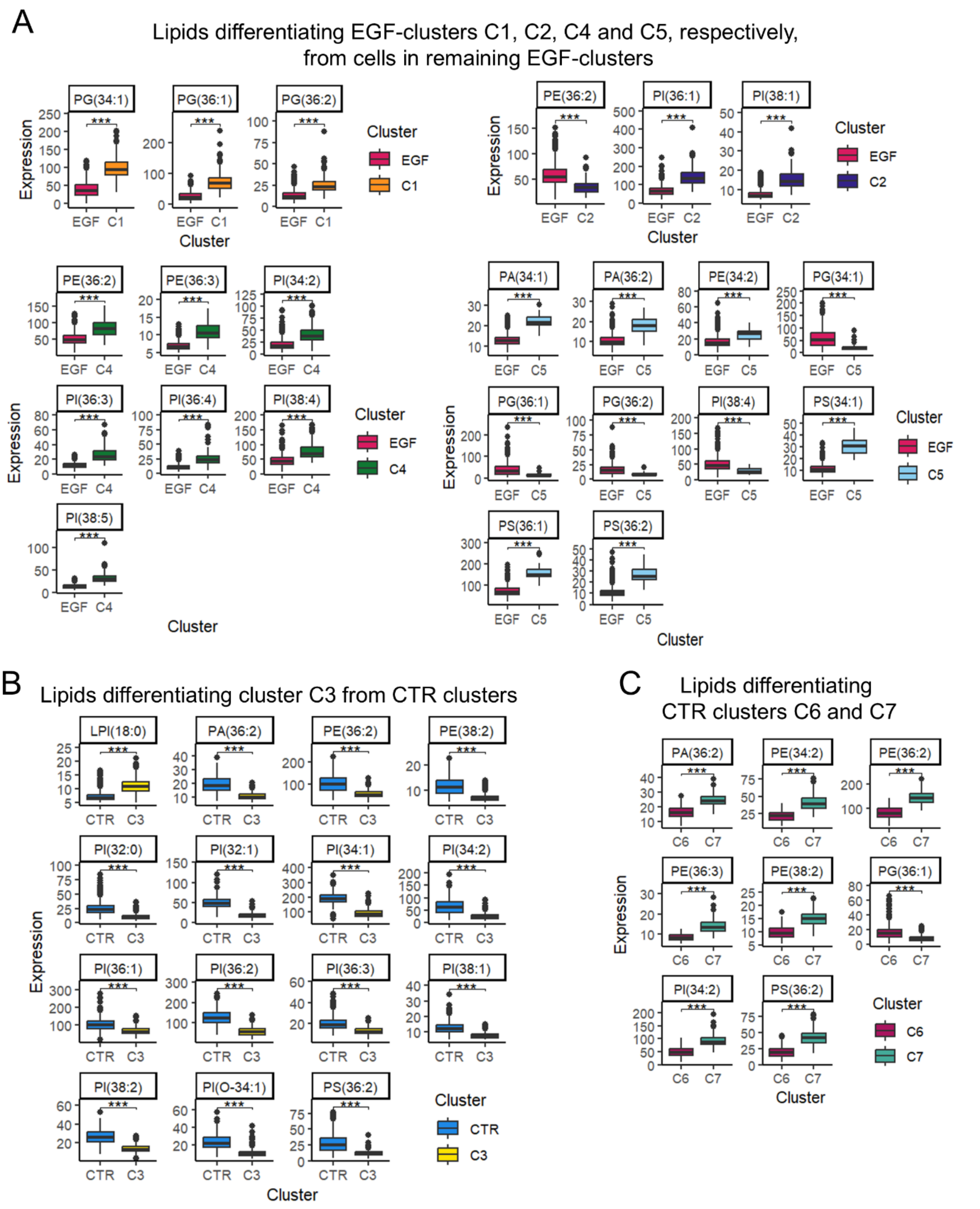

*Figure S2:Boxplots displaying levels of lipids highlighted in Figure 4A. A:Lipids differentiating EGF-cluster C3 from pooled CTR clusters (yellow boxes in figure4A). B: Lipids differentiating CTR clusters C6 and C7 (blue boxes in figure 4A). C: Lipids differentiating each of EGF-clusters C1, C2, C4 and C5 from remaining EGF-clusters, pooled (black boxes in figure 4A). The boxes represent the interquartile range (IQR) while the error bars represent Q1 - 1.5*IQR and Q3 +1.5*IQR respectively. P-values calculated using two-sample t tests and corrected using the Bonferroni approach. Significance levels: p<0.05: "*", p<0.01: "**", p<0.001: "***" . All plotted lipid comparisons fulfill the following: fold change >1.5 or below 0.667, p-value <0.001 and non-overlapping interquartile range (IQR).*

4. Gene expression analysis of bulk RNA sequencing data

Table S3: Results of Gene set enrichment analysis against the MSigDB hallmark EMT gene set for each EGF-treatment against the control. Related to results section on Gene expression. Input lists were significantly (p.adj <0.05) differentially expressed and ranked according to fold change. Significance levels: p<0.05: "", p<0.01: "**", p<0.001: "***".*

Gene list input	P-value	Significance level	log2error	Enrichment Score	Normalized enrichment score
12h vs CTR	4.22e-14	***	0.965	0.573	2.023
24h vs CTR	2.66-10	***	0.814	0.513	1.833
48h vs CTR	2.42e-06	***	0.627	0.465	1.652
72h vs CTR	2.28e-11	***	0.863	0.527	1.884

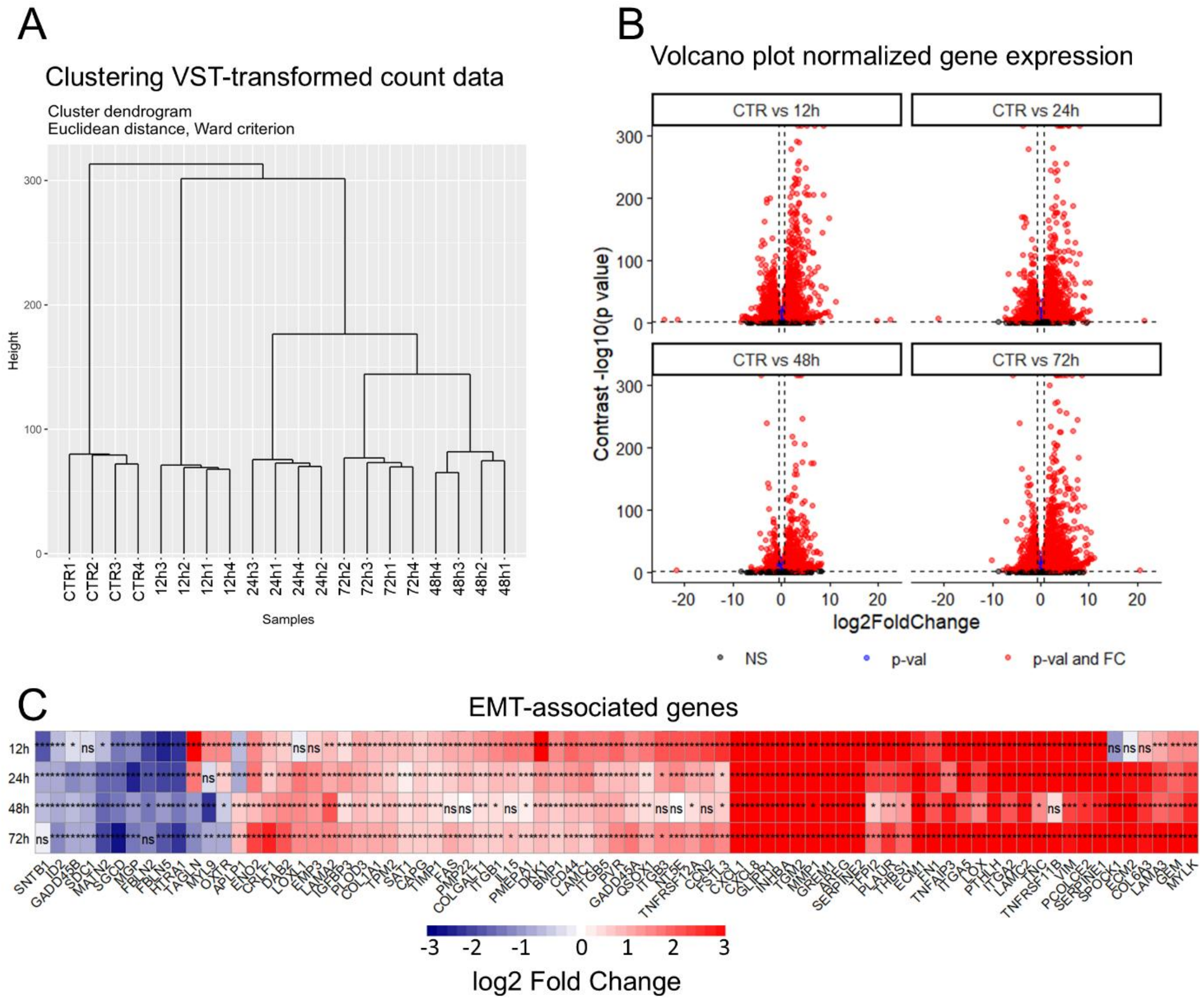

Figure S3: Gene expression changes in response to EGF in MDA-MB-468. Related to Results section on Gene expression. A: Dendrogram displaying clustering of count data after Variance Stabilizing Transformation (VST). The dendrogram was generated as part of the SARTools pipeline.[S1] B: Volcano plots of log2 Fold Changes (FC) and log-transformed adjusted p-values in each EGF-treatment compared to control (CTR), using a p-value cut-off of 0.05 and

fold change cut-off of +/- log2(1.5). C: Log2 Fold Change and significance levels of EMT Hallmark genes that are differentially expressed in at least three of the EGF-treatments compared to CTR. P-values for RNA-seq data were calculated in the SARTools package using a Benjamin-Hochberg-corrected Walds test. Significance levels: p<0.05: "", p<0.01: "**", p<0.001: "***"*

5. Gene expression analysis of single-cell RNA sequencing data

Sc RNA seq EMT inductions # times gene measured	Gene	Bulk RNA seq log2 fc MDA-MB-468 EGF 1d vs CTR	Sc RNA seq # EMT inductions log 2 fc > 0.585 1d vs ctr	Sc RNA seq # EMT inductions log 2 fc < -0.585 1d vs ctr	Bulk RNA seq log2 fc MDA-MB-468 EGF 3d vs CTR	Sc RNA seq # EMT inductions log 2 fc > 0.585 3d vs ctr	Sc RNA seq # EMT inductions log 2 fc < -0.585 3d vs ctr
12	AGPAT2	ns			*		
12	AGPAT5	***		2	***		1
12	CEPT1	***	1		***	2	
12	DGKA	***	1		***	1	
12	DGKD	***			***	1	
12	DGKE	***		1	***		1
12	DGKH	***			***		
12	DGKQ	***		1	*		1
12	DGKZ	ns			ns		
12	ETNK1	**			ns		
12	FADS1	**			*		
12	FADS2	***			***		
9	LIPH	***	1		***		1
12	LPCAT1	ns	1	1	**	1	1
12	LPCAT3	***			***		
12	LPCAT4	***			***		
12	LPIN2	**	1		**		
12	LPIN3	ns			**		
12	MBOAT2	***	4		***	4	
12	PGS1	*			ns	1	
12	PISD	ns			**		
7	PLA2G10	ns		3	*	1	2
12	PLA2G12A	***			ns		
12	PLA2G15	ns	1		ns	3	1
4	PLA2G4C	*	2		***	2	
2	PLA2G6	ns			ns		
12	PLD1	***			***		
12	PLD2	ns			ns		
12	SCD	***	1	1	**	1	1

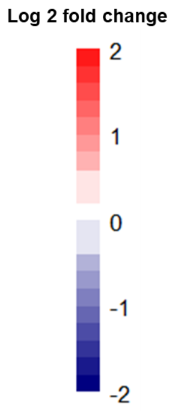

Figure S4: Consensus of EMT-induced regulation of glycerophospholipid biosynthesis genes and genes involved in fatty acid desaturation. Related to Results section on gene expression. Expression after 1 day and 3 days of EMT induction compared to control. Log2 fold change and significance levels for bulk RNAseq data is visualized as in Figure 6B. Sc RNA seq columns show counts of number of EMT inductions where each gene is significantly (p.adj<0.05) up- or downregulated with log2 fold change above 0.585 or below -0.585, respectively. The sc RNAseq data was acquired from Cook and Vanderhyden.[S2] P-values calculated using the Wilcoxon rank sum test and adjusted using Holm p-value adjustment. Significance levels: p<0.05: "", p<0.01: "**", p<0.001: "***"*

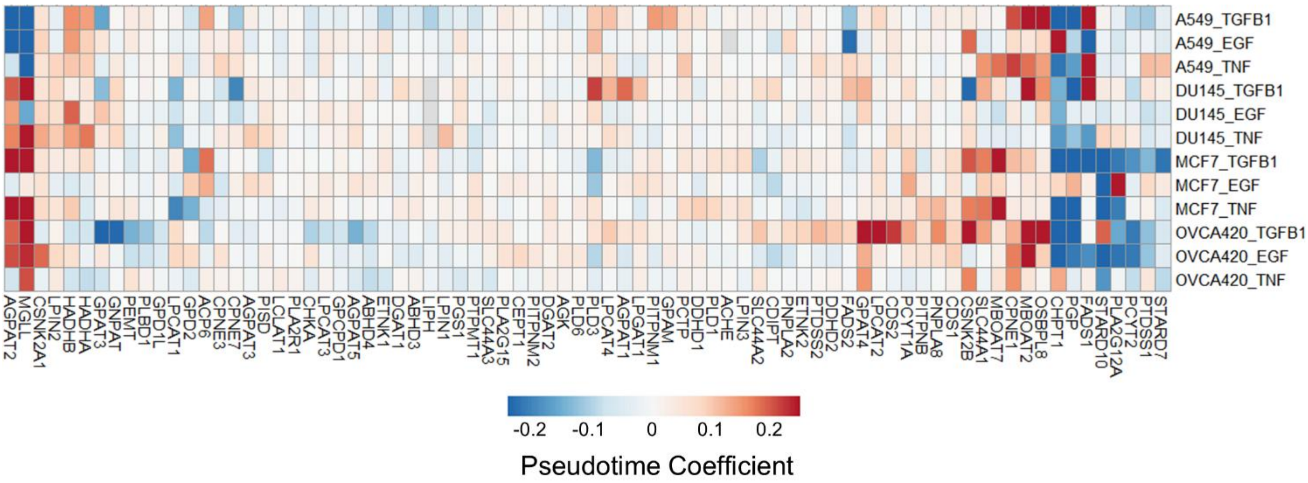

Figure S5: Heatmap of gene expression changes of genes involved in glycerophospholipid biosynthesis and FADS1/2 during EMT-induction in four separate cell lines and three different modes of EMT-induction. The heatmap is colored according to the pseudotime beta coefficient of each genes' linear model. The data was acquired from Cook and Vanderhyden.[S2]

Bibliography

[S1] Varet, H., Brillet-Guéguen, L., Coppée, J.-Y., and Dillies, M.-A. (2016). SARTools: A DESeq2- and EdgeR-Based R Pipeline for Comprehensive Differential Analysis of RNA-Seq Data. PLoS One *11*, e0157022.

[S2] Cook, D.P., and Vanderhyden, B.C. (2020). Context specificity of the EMT transcriptional response. Nat. Commun. *11*, 2142. https://doi.org/10.1038/s41467-020-16066-2.
